# Coverage of HIV Pre-Exposure Prophylaxis (PrEP) Within the Active Duty U.S. Military, 2023

**Published:** 2024-03-20

**Authors:** James D. Mancuso, Anwar E. Ahmed

**Affiliations:** 1 Uniformed Services University Department of Preventive Medicine and Biostatistics

## Abstract

Estimates of HIV pre-exposure prophylaxis (PrEP) coverage in the U.S. military, defined as the proportion of the persons taking HIV PrEP out of the estimated number of persons who had indications for it, have never been published. The objective of this study was to provide an estimate of HIV PrEP coverage comparable to U.S. civilian estimates. The population with indications for HIV PrEP was obtained from the Department of Defense 2018 Health Related Behaviors Survey, a stratified random sample of members of all military service branches. The military PrEP coverage estimate of 31.6% in 2023 was lower than the national U.S. estimate of 36.0% in 2022. Among the military population of men who have sex with men (MSM), an estimated 24.6% of service members had indications for PrEP, similar to the national estimate of 24.7%. MSM comprised 66% of all military service members with HIV PrEP indications, compared to 40% in the U.S. general population. The U.S. military should continue deliberate, sustained, and effective actions to address sexual health inequities among MSM, aligned and coordinated with societal efforts including improved coverage of HIV PrEP to prevent HIV transmission.

**What are the new findings?:**

Coverage of HIV PrEP among active duty military service members with indications for its use increased to 31.6% in 2023, which was lower than the national U.S. estimate of 36.0% in 2022. Men who have sex with men constituted 66% of all U.S. military service members with HIV PrEP indications.

**What is the impact on readiness and force health protection?:**

HIV PrEP may be underutilized within the U.S. military despite its effectiveness in preventing HIV transmission and its resultant improvements in morbidity, health care costs, and impacts on deployability and attrition. Men who have sex with men are the most important group for which the military should promote HIV PrEP use.

## 1 BACKGROUND

Despite effective interventions to diagnose, treat, and prevent the transmission of the human immunodeficiency virus (HIV), 36,136 new infections occurred in the U.S. in 2021.^1^ Male-to-male sexual contact accounted for 67% of these infections, and 56% of those infected were ages 13 to 34, 40% were non-Hispanic Black, and 29% were Hispanic. Among the important interventions to prevent HIV transmission is pre-exposure prophylaxis (PrEP) with emtricitabine and tenofovir disoproxil fumarate or other approved regimens.

PrEP has been shown to be safe, as well as demonstrating an average effectiveness of 75% in reducing risk of HIV transmission among patients at high risk for HIV acquisition, although its effectiveness may be more or less than 75% depending on patient adherence.^2^ Since 2011,^3^ the use of PrEP has been recommended for this purpose among persons at high risk for HIV transmission by the U.S. Centers for Disease Control and Prevention (CDC).^4^ It is a critical component of the National HIV/AIDS Strategy for the United States^5^ and the Ending the HIV Epidemic in the U.S. initiative.^6^ Despite its importance in HIV prevention, the CDC estimated that only 36.0% of persons for whom HIV PrEP use was indicated were actually taking it in 2022,^7^ although this figure had increased from 12.6% in 2017.^8^

U.S. Department of Defense (DOD) policy offers HIV PrEP in accordance with CDC guidance and as directed in national objectives.^9,10^ A prior study found that, from February 2014 to June 2016, an estimated 769 active duty service members had taken HIV PrEP, out of an indirectly estimated (using civilian data) population of 12,000 with indications.^11^

PrEP coverage in the U.S. military, defined as the proportion of the persons taking PrEP out of the estimated number of persons with indications for PrEP, has never been directly estimated using military data. An updated estimate of 4,495 service members taking PrEP in 2023 is provided in this month’s issue of *MSMR*.^12^ The objective of this study was to provide a direct estimate of the denominator, or population of service members with PrEP indications, to provide an estimate of PrEP coverage comparable to U.S. civilian estimates.

## 2 METHODS

The population at risk was obtained from the DOD 2018 Health Related Behaviors Survey (HRBS), which is comparable to the active duty population in 2023.^13^ The HRBS study population was a stratified random sample of members of all military service branches—Army, Navy, Air Force, Marine Corps, and Coast Guard.^14^ Of 199,996 invited eligible active duty service members, the overall weighted response rate was 9.6%.^14^

This study employed a total sampling frame of 1,357,219 active duty service members, and this was segmented into 50 strata based on the interaction of service branch (5 categories), pay grade (5 categories), and sex. All analyses were performed using SAS 9.4 (SAS Institute Inc., Cary, NC). Analyses accounted for survey weights to generate estimates representative of the active duty U.S. military population.

Estimates of the total U.S. military population of men who have sex with men (MSM), intravenous drug users (IVDUs), and sexually-active heterosexual men and women were obtained using HRBS questions 4 and 48 (MSM), 40c (IVDUs), and 46, 48, 49 (sexually-active heterosexuals).^14^ Estimates of the population of MSM who had indications for PrEP were also obtained from the HRBS, using the same methods used previously by CDC.^15^

MSM were considered as having indications for PrEP if they reported sex with 2 or more men in the past 12 months and any condom-less sex or a sexually-transmitted infection (STI) within the past 12 months (HRBS questions 46, 47, 54).^14^ Estimates of the population of IVDUs and heterosexual men and women with indications for PrEP were obtained by multiplying these populations from the HRBS by the proportion estimated to have indications for PrEP among civilian populations in 2018,^15^ as no similar questions pertaining to these indications for these groups were included in the HRBS. Estimates of MSM, heterosexual, and IVDU risks were independent of one another.

Both stratified and aggregate estimates of PrEP coverage in the U.S. active duty military are reported. Methods used to obtain national civilian estimates have been described previously.^7,15^ Institutional review was performed by the Uniformed Services University of the Health Sciences, Bethesda, MD.

## 3 RESULTS

As seen in the **Table**, there were an estimated 38,341 MSM, 9,599 IVDUs, and 1,138,259 sexually-active heterosexual men and women in the active duty U.S. military population. These 3 subpopulations comprised, respectively, 2.8%, 0.7%, and 83.9% of the total estimated active duty population of 1,357,219 estimated by the HRBS (data not shown).^14^

Among MSM, 9,441 (24.6%) reported 1 or more indications for PrEP. There were also an estimated 1,776 IVDUs, 1,908 heterosexual men, and 1,106 heterosexual women with PrEP indications. These results generated an estimate of 14,231 total service members with PrEP indications. As 4,495 active duty service members were estimated to have been prescribed PrEP in 2023,^12^ this resulted in an estimated PrEP coverage of 31.6%. Of note, 66% of military service members with HIV PrEP indications were MSM, compared to only 40% of the U.S. population. The corresponding figures from the U.S. general population are also shown in the **Table**.

## 4 DISCUSSION

This report estimates that PrEP coverage among active duty U.S. military service members in 2023 was 31.6%. The DOD estimate of PrEP coverage of 31.6% in 2023 was lower than the 2022 national U.S. estimate of 36.0%. Among those with PrEP indications, MSM comprised 66% of U.S. service members but only 40% of the U.S. population. The difference between these populations is largely attributable to the larger proportion of males in the U.S. military.

The comparison of PrEP coverage may be limited by demographic differences, with the military comprised of a younger and more racially and ethnically diverse population than the U.S. as a whole.^16^ Differences in health-related and other behaviors between these populations may also limit these comparisons.^14^ Additionally, the low response rate and the ‘healthy warrior effect’ may limit comparisons between military and civilian populations in this study due to selection bias.^14^

As with any self-reported data, health behaviors may have been misclassified due to concerns about social desirability, stigma, or other factors. The proportions of IVDUs and sexually-active heterosexual service members could not be directly estimated because the HRBS does not contain the necessary data for these calculations, which may have resulted in misclassification. For example, the CDC considers only those IVDUs who “have injected any assessed drug during the past 12 months and used a needle that had previously been used by another person” as having an indication for PrEP.^15^ The estimate of U.S. military members who were IVDUs was obtained from a question that also included other illegal drug use not limited to injection (e.g., cocaine, LSD, ecstasy, PCP), with no assessment of needle reuse.^14^ The U.S. military also performs routine testing for IV and other illegal drug use. These factors likely resulted in an overestimation of both service members who were IVDUs and those who had indications for PrEP, although this would have had only a small impact on the results of this report.

Similarly, the CDC only considered heterosexuals as having indications for PrEP if they “reported sex with two or more opposite sex partners and either 1) sex with an HIV-infected partner or 2) any condom-less sex in the last 4 weeks and sex with a high-risk partner in the past 12 months.”^15^ High-risk partners were defined as “persons who inject drugs or (for women) male partners known to also have sex with men (behaviorally bisexual).” This information on the behaviors of heterosexual service members and their sexual partners’ risk factors was likewise not available from the HRBS nor any other military source. Both national and DOD estimates may underestimate the heterosexual population with PrEP indications due to changing indications for PrEP in the 2021 U.S. Public Health Service and CDC guidelines, as neither included the new indication of a bacterial sexually-transmitted infection within the past 6 months.^4,7^ Similar to the CDC’s estimates, the groups studied in this report may not be mutually exclusive, resulting in a small overestimation in both estimates for the total population with PrEP indications. There is an expected small underestimation of the total active duty population prescribed PrEP, as it excludes activated Guard and Reserve service members.

Most importantly, service members may have sought HIV PrEP beyond direct or private sector health care provided by the Military Health System, instead seeking it at a local health department or privately-funded clinic serving the MSM community. If such care was not reimbursed by the military, the DOD would have no record of it, resulting in underestimates of HIV PrEP coverage in this report.

HIV degrades military readiness through the direct and indirect costs of HIV-associated health care and through deployment limitations and attrition of personnel with critical military occupational skills and experience.^18,19^ This study suggests that HIV PrEP use in the active duty military remains lower than in the U.S. population, and may be underutilized. This difference in use may be due to health care system factors, cultural factors, or demographic and behavioral differences between the U.S. military and civilian populations, although differences due to misclassification of PrEP use due to non-DOD health care utilization or other biases cannot be excluded. It further suggests that MSM in the U.S. military have similar behaviors resulting in HIV PrEP indications as MSM in the civilian population.

The high proportion (66%) of MSM among those with indications for HIV PrEP in the U.S. military suggests that this is the most important group in which the military should promote PrEP use. The U.S. military should continue deliberate, sustained, and effective actions to address sexual health inequities among MSM, commensurate and coordinated with societal efforts, that include improved HIV PrEP coverage to prevent HIV transmission.

## Figures and Tables

**Table.** Populations with PrEP Indications and Prescriptions, U.S. General Population and U.S. Military

	**U.S. General Population**					**U.S. Military**				
	**Total Population^a^**	**PrEP Indications^a^**		**Prescribed PrEP in 2022^b^**		**Total Population^c^**	**PrEP Indications**		**Prescribed PrEP in 2022**	
	No.	No.	%	No.	%	No.	No.^e^	%^d^	No.	%
**MSM**	1,991,903	492,000	24.7%			38,341	9,441	24.6%		
**IVDUs**	621,622	115,000	18.5%			9,599	1,776	18.5%		
**Sexually-active heterosexuals**	156,000,000	624,000	0.4%			1,138,259		0.4%		
**Men**	78,500,000	157,000	0.2%			953,939	1,908	0.2%		
**Women**	78,000,000	468,000	0.6%			184,320	1,106	0.6%		
**Total**	158,613,524	1,216,210^g^	0.8%	437,666	36.0%	1,186,199	14,231	1.2%	4,495^f^	31.6%

Abbreviations: PrEP, HIV pre-exposure prophylaxis; No., number; MSM, men who have sex with men; IVDUs, intravenous drug users; HRBS, Health Related Behaviors Survey.

^a^Reference 11

^b^Reference 7

^c^Derived from HRBS questions 4 and 48 (MSM); 40c (IVDUs); and 46, 48, 49 (sexually-active heterosexuals).

^d^Derived from HRBS questions 46, 47, 54 (MSM) or reference 11 (IVDU and sexually-active heterosexuals).

^e^Obtained by multiplying the population in each category and the percent with PrEP indications.

^f^Reference 12

^g^Subgroup frequencies do not exactly match total population frequency due to minor annual variation between references 7 and 11.

## AUTHOR AFFILIATIONS

Uniformed Services University Department of Preventive Medicine and Biostatistics: Dr. Mancuso and Dr. Ahmed

## References

[1] Centers for Disease Control and Prevention. HIV Surveillance Report: Diagnoses of HIV Infection in the United States and Dependent Areas, 2021..

[2] Murchu EO, Marshall L, Teljeur C (2022). Oral pre-exposure prophylaxis (PrEP) to prevent HIV: a systematic review and meta-analysis of clinical effectiveness, safety, adherence and risk compensation in all populations.. BMJ Open..

[3] Centers for Disease Control and Prevention. (2011). Interim guidance: preexposure prophylaxis for the prevention of HIV infection in men who have sex with men.. MMWR Morb Mortal Wkly Rep..

[4] US Public Health Service. *Preexposure Prophylaxis for the Prevention of HIV Infection in the United States–2021 Update: A Clinical Practice Guideline.*.

[5] The White House. *National HIV/AIDS Strategy for the United States 2022-2025.*.

[6] Division of HIV Prevention. Ending the HIV Epidemic in the U.S. Centers for Disease Control and Prevention..

[7] Centers for Disease Control and Prevention. (2023). Core indicators for monitoring the Ending the HIV Epidemic Initiative (preliminary data): National HIV Surveillance System Data Reported Through March 2023; and preexposure prophylaxis (PrEP) data reported through December 2022.. HIV Surveillance Data Tables..

[8] Centers for Disease Control and Prevention. *HIV Surveillance Data Tables (early release): Core Indicators for Monitoring the Ending the HIV Epidemic Initiative: Data Reported Through December 2019.*.

[9] Defense Health Agency. Defense Health Agency Procedural Instruction 6025.29: Provision of Human Immunodeficiency Virus (HIV) Pre-Exposure Prophylaxis (PrEP) for Persons at High Risk of Acquiring HIV Infection..

[10] The White House. *National HIV/AIDS Strategy Federal Implementation Plan. Updated Aug. 21, 2023.*.

[11] Blaylock JM, Hakre S, Okulicz JF (2018). HIV preexposure prophylaxis in the U.S. military services–2014-2016.. MMWR Morb Mortal Wkly Rep..

[12] Eick-Cost AA, Mabila SL, Ying S (2024). Surveillance snapshot: HIV pre-exposure prophylaxis (PrEP) prescriptions in the active component of the U.S. Military, 2023.. MSMR..

[13] Office of the Deputy Assistant Secretary of Defense for Military Community and Family Policy. *2021 Demographics: Profile of the Military Community. Department of Defense.*.

[14] Meadows SO, Engel CC, Collins RL 2018 Department of Defense Health Related Behaviors Survey (HRBS)..

[15] Smith DK, Van Handel M, Wolitski RJ (2015). Vital signs: estimated percentages and numbers of adults with indications for preexposure prophylaxis to prevent HIV acquisition–United States, 2015.. MMWR Morb Mortal Wkly Rep..

[16] Military OneSource. *2019 Demographics Profile of the Military Community.*.

[17] Hoover KW, Zhu W, Gant ZC (2022). HIV Services and outcomes during the COVID-19 pandemic–United States, 2019-2021.. MMWR Morb Mortal Wkly Rep..

[18] Cavanaugh JS, Murray CK, Chang D, Ake JA (2023). The Purpose and Impact of the U.S. Military HIV Research Program.. Joint Force Quarterly..

[19] Under Secretary of Defense for Personnel and Readiness. DOD Instruction 6485.01: Human Immunodeficiency Virus (HIV) in Military Service Members..

